# Supplementary figures and images for: Enhanced contact investigations for nine early travel-related cases of SARS-CoV-2 in the United States
Source: PLoS One. 2020 Sep 2;15(9):e0238342. doi: 10.1371/journal.pone.0238342 (PMC7467265; doi:10.1371/journal.pone.0238342)

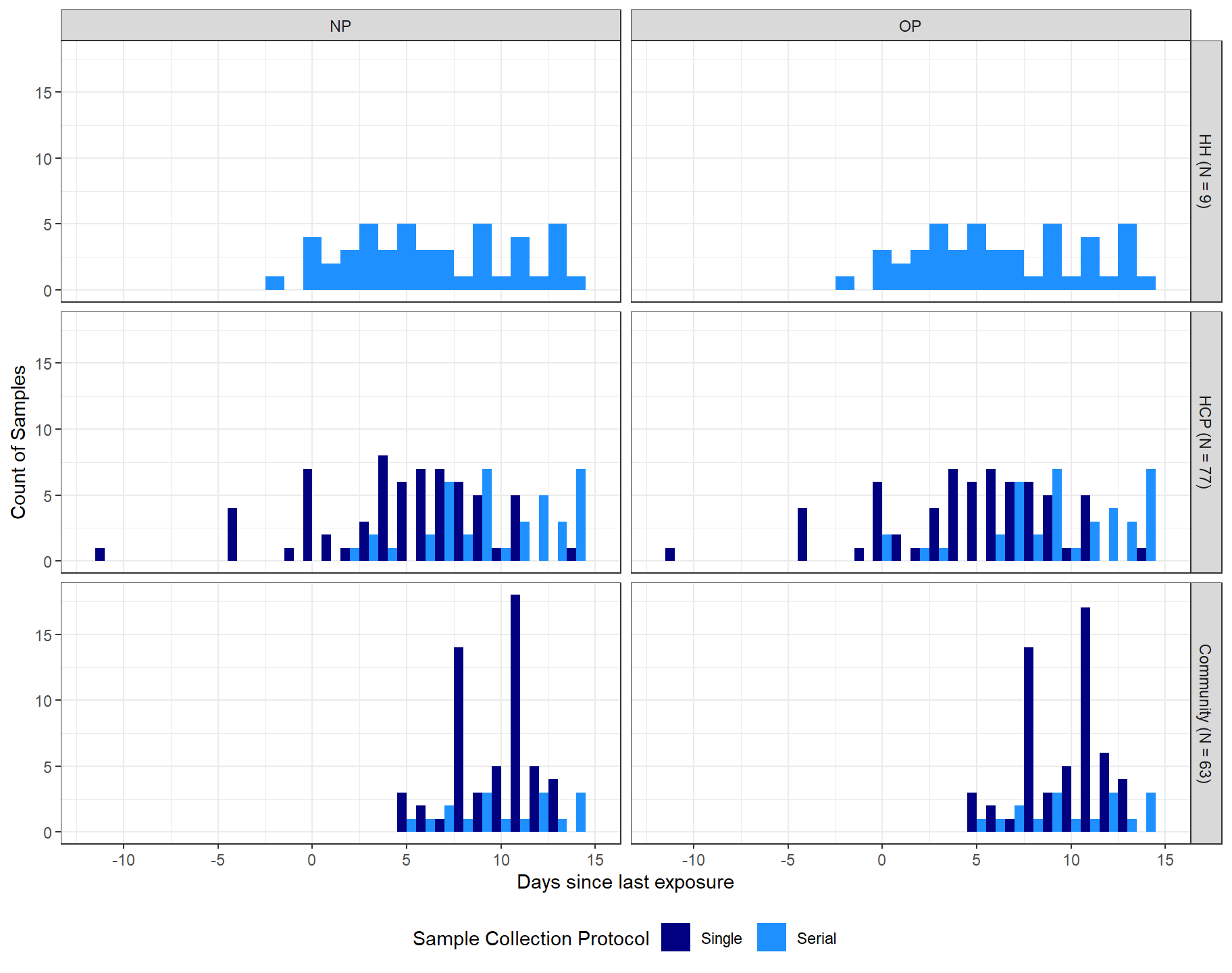

Supplement: S1 Fig — Specimens (N = 448) collected from 149 contacts with known exposure periods are shown by the days following last exposure to the confirmed COVID-19 patient on the x axis. Excludes specimens from five household contacts with ongoing household exposure after the diagnosis of the travel-associated case patient, one household member diagnosed concurrently as the travel-associated case patient, and four community contacts without a known exposure period or date of collection. Specimens from contacts submitting only a single set of respiratory specimens are shown in navy, while specimens from those submitting multiple sets of respiratory specimens are shown in lighter blue. Household contacts (HH) are shown in the first row (excluding 5 who were co-habiting with the case patient following diagnosis and 1 who was concurrently diagnosed), Healthcare Personnel (HCP) contacts are shown in the second row, and community contacts are shown in the bottom row. The first column shows the number of nasopharyngeal (NP) swabs collected and tested, and the second column shows the number of oropharyngeal (OP) swabs collected and tested. Some HH members and HCP have specimens collected at negative days from last exposure—these represent specimens collected during a period of ongoing exposure to the COVID-19 patient. Samples collected relative to the first day of exposure are shown in S1 Fig. Only the initial specimens are shown for the secondary cases, as these specimens tested positive. (TIFF) [file pone.0238342.s001.tiff]

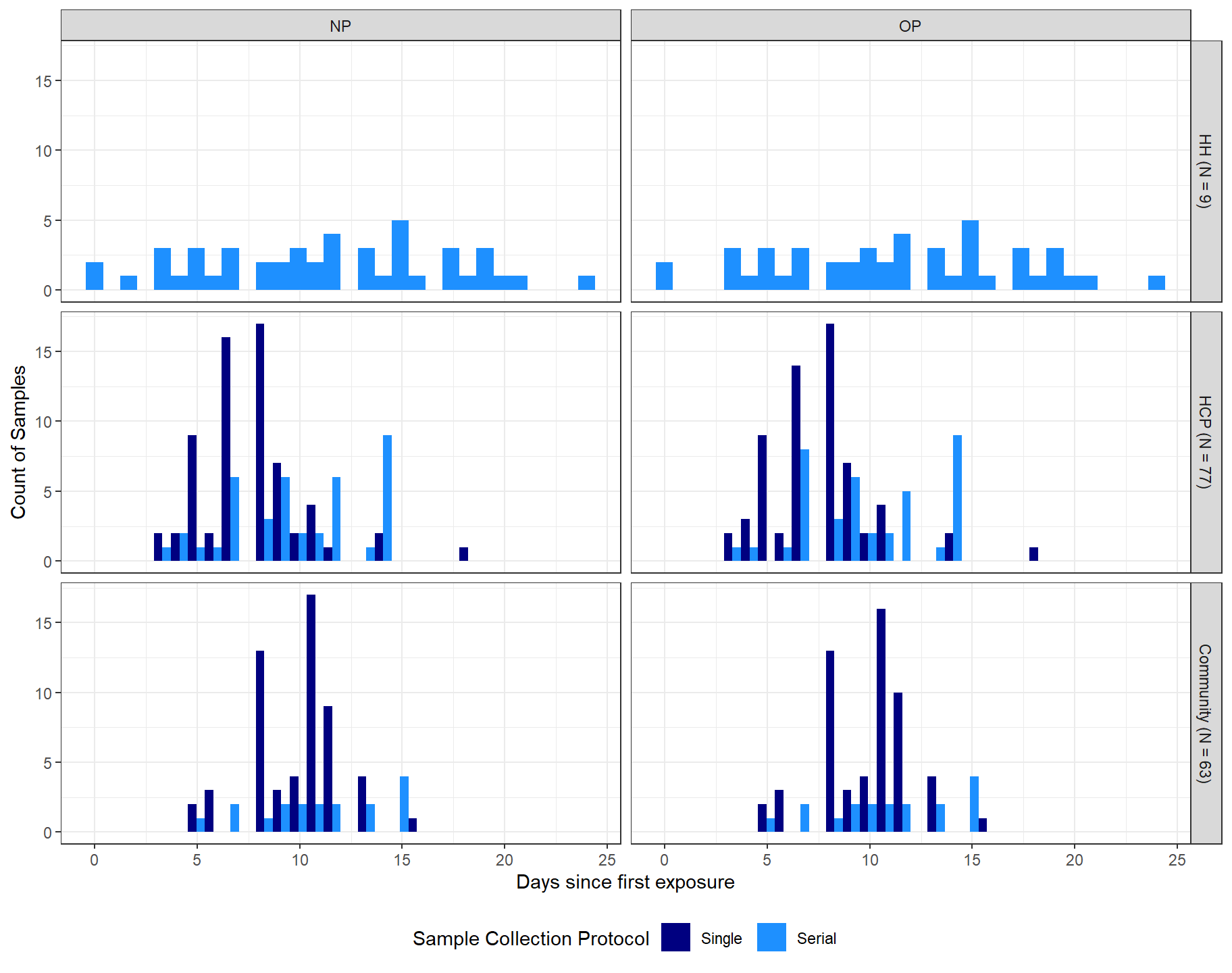

Supplement: S2 Fig — Specimens (N = 448) collected from 149 contacts with known exposure periods are shown by the days following first exposure to the confirmed COVID-19 patient on the x axis. Excludes specimens from five household contacts with ongoing household exposure after the diagnosis of the travel-associated case patient, one household member diagnosed concurrently as the travel-associated case patient, and four community contacts without a known exposure period or date of collection. Specimens from contacts submitting only a single set of respiratory specimens are shown in navy, while specimens from those submitting multiple sets of respiratory specimens are shown in lighter blue. Household contacts (HH) are shown in the first row, Healthcare Personnel (HCP) contacts are shown in the second row, and community contacts are shown in the bottom row. The first column shows the number of nasopharyngeal (NP) swabs collected and tested, and the second column shows the number of oropharyngeal (OP) swabs collected and tested. Only the initial specimens are shown for the secondary cases, as these specimens tested positive. (TIFF) [file pone.0238342.s002.tiff]

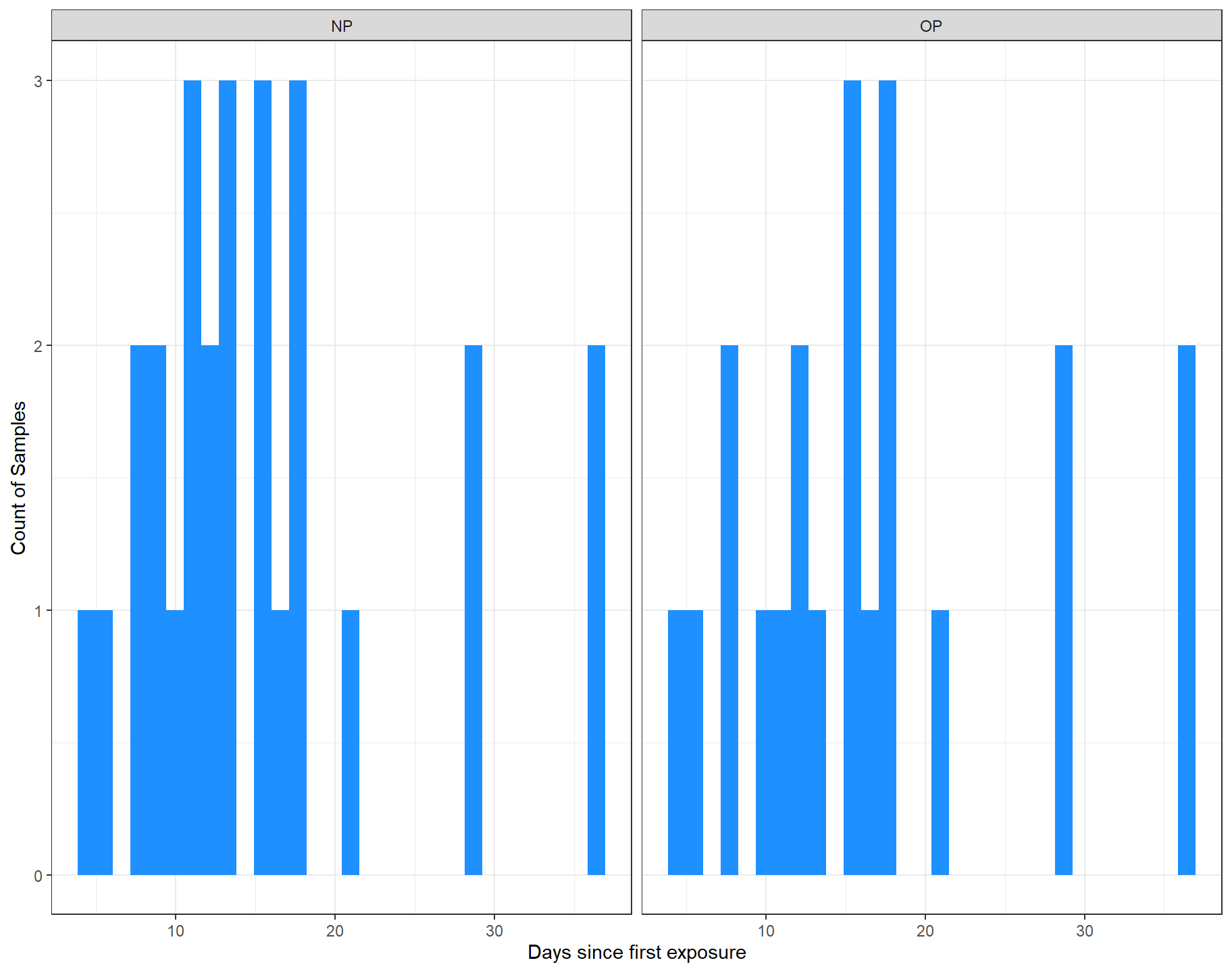

Supplement: S3 Fig — Specimens (N = 48) collected from 5 contacts who were co-habiting with a COVID-19 patient following the travel-associated case patient’s diagnosis are shown by the days following first exposure to the confirmed COVID-19 patient on the x axis. The first column shows the number of nasopharyngeal (NP) swabs collected and tested, and the second column shows the number of oropharyngeal (OP) swabs collected and tested. (TIFF) [file pone.0238342.s003.tiff]
